# Knockdown of LncRNA SCAMP1 suppressed malignant biological behaviours of glioma cells via modulating miR‐499a‐5p/LMX1A/NLRC5 pathway

**DOI:** 10.1111/jcmm.14362

**Published:** 2019-06-17

**Authors:** Zheqi Zong, Yichen Song, Yixue Xue, Xuelei Ruan, Xiaobai Liu, Chunqing Yang, Jian Zheng, Shuo Cao, Zhen Li, Yunhui Liu

**Affiliations:** ^1^ Department of Neurosurgery Shengjing Hospital of China Medical University Shenyang China; ^2^ Liaoning Clinical Medical Research Center in Nervous System Disease Shenyang China; ^3^ Key Laboratory of Neuro‐oncology in Liaoning Province Shenyang China; ^4^ Department of Neurobiology, School of Life Sciences China Medical University Shenyang China; ^5^ Key Laboratory of Cell Biology, Ministry of Public Health of China China Medical University Shenyang China; ^6^ Key Laboratory of Medical Cell Biology, Ministry of Education of China China Medical University Shenyang China

**Keywords:** glioma, LMX1A, long non‐coding RNAs, miR‐499a‐5p, NLRC5, SCAMP1

## Abstract

Dysregulation of long non‐coding RNAs (lncRNAs) confirm that it plays a crucial role in tumourigenesis and malignant progression of glioma. The present study demonstrated that LncRNA secretory carrier membrane protein 1 (SCAMP1) was up‐regulated and functioned as an oncogene in glioma cells. In addition, miR‐499a‐5p was down‐regulated meanwhile exerted tumour‐suppressive function in glioma cells. Subsequently, inhibition of SCAMP1 significantly restrained the cell proliferation, migration and invasion, as well as promoted apoptosis by acting as a molecular sponge of miR‐499a‐5p. Transcription factor LIM homeobox transcription factor 1, alpha (LMX1A) was overexpressed in glioma tissues and cells. Moreover, miR‐499a‐5p targeted LMX1A 3′‐UTR in a sequence‐specific manner. Hence, down‐regulation of SCAMP1 remarkably reduced the expression level of LMX1A, indicating that LMX1A participated in miR‐499a‐5p‐induced tumour‐suppressive effects on glioma cells. Furthermore, knockdown of LMX1A decreased NLR family, CARD domain containing 5 (NLRC5) mRNA and protein expression levels through directly binding to the NLRC5 promoter region. Down‐regulation of NLRC5 obviously inhibited malignant biological behaviours of glioma cells through attenuating the activity of Wnt/β‐catenin signalling pathway. In conclusion, our study clarifies that SCAMP1/miR‐499a‐5p/LMX1A/NLRC5 axis plays a critical role in modulating malignant progression of glioma cells, which provide a novel therapeutic strategy for glioma treatment.

## 1 INTRODUCTION

Glioma, which represents approximately 47.1% of all CNS malignant tumours, is considered to be the most lethal primary brain tumour.^1^, ^2^ Despite recent advances in surgery, chemotherapy and radiotherapy, the prognosis of glioma has not improved significantly, whereas patients with glioma still present a dismal outcome with a poor median survival time of only 12‐15 months.^3^, ^4^ Therefore, precise gene‐targeted therapy is expected to become an effective therapeutic strategy for glioma.

Numerous evidence indicate that long non‐coding RNAs (lncRNAs) act as an important part in controlling the malignant progression of human tumours at gene expression level in almost every aspect of biological processes.^5^, ^6^ Secretory carrier membrane protein 1 (SCAMP1) functions as a carrier and participates in post‐Golgi recycling pathway.^7^ Accumulating reports have suggested that dysregulation of SCAMP1 is intimately associated with the development of various tumours.^8^, ^9^, ^10^ However, little is known about the functions of lncRNA SCAMP1 in glioma. MicroRNAs (miRNAs) have demonstrated that they exert crucial modulatory roles on the tumourigenesis and the progression of glioma.^11^, ^12^, ^13^ miR‐499a‐5p, also known as miR‐499‐5p or miR‐499, was found to act as a tumour suppressor in osteosarcoma and oesophageal carcinoma.^14^, ^15^ However, the potential role of miR‐499a‐5p in glioma has not been reported.

Transcription factor LIM homeobox transcription factor 1, alpha (LMX1A) located in 1q24.1 is a family member of LIM homeobox‐containing genes that encode LIM‐homeodomains.^16^ Increasing researches have revealed that LMX1A is a key participant in modulating biological behaviours of varied human tumours.^17^, ^18^, ^19^ Overexpression of LMX1A was associated with shorter survival and advanced clinicopathologic parameters in pancreatic ductal adenocarcinomas.^18^ Moreover, LMX1A was reported to produce oncogenic effects on tumour development of cervical cancer.^19^ However, the mechanisms underlying the role of LMX1A in glioma remain unclarified. NLR family, CARD domain containing 5 (NLRC5) is a member of the NLR protein family, which plays a critical role in cytokine response and regulation of immune responses.^20^ Growing studies have shown that NLRC5 is involved in multiple human tumour progression.^21^, ^22^ For instance, overexpression of NLRC5 promoted the development of hepatocellular carcinoma by targeting the Wnt/β‐catenin signalling pathway.^22^ However, whether NLRC5 is involved in the malignant progression of glioma was still unclear.

In our study, the expression levels of SCAMP1, miR‐499a‐5p, LMX1A and NLRC5 were investigated in glioma tissues and cell lines, meanwhile, the effects on regulating malignant progression of glioma and cross‐talk among SCAMP1, miR‐499a‐5p, LMX1A and NLRC5 were completely clarified. Our findings may provide a novel therapeutic strategy for glioma.

## 2 MATERIALS AND METHODS

### 2.1 Clinical specimens

A total of 31 glioma specimens and six normal brain tissues (NBTs) were obtained from the Department of Neurosurgery, Shengjing Hospital of China Medical University. NBTs were the rejected materials obtained from surgeries of severe intracerebral haemorrhage, brain trauma and epilepsy. After surgical resection, all human tissues were immediately frozen in liquid nitrogen for long‐term preservation. Glioma tissues were classified into four grades according to the WHO classification of tumours in the CNS, then divided into two groups: low‐grade glioma group (Grade I–II, 15) and high‐grade glioma group (Group III–IV, 16). This research had obtained approval of Shengjing Hospital Ethics Committee.

### 2.2 Cell lines and cultures

Normal human astrocyte (NHA) cells were purchased from Sciencell Research Laboratories (Carlsbad, CA) and human glioma U87, U251 and HEK293T cells were purchased from Shanghai Institutes for Biological Sciences Cell Resource Center. NHA cells grown in astrocyte medium and U87, U251, HEK293T cells were cultured in Dulbecco's modified Eagle medium (DMEM)/high glucose mixed with 10% foetal bovine serum (Gibco, Carlsbad, CA). All cells were maintained in a humidified incubator at 37°C with 5% CO2.

### 2.3 Cell transfection

Short‐hairpin RNA (shRNA) against SCAMP1, LMX1A and NLRC5 gene, as well as their non‐targeting sequences were constructed in pGPU6/GFP/Neo vector from GenePharama (Shanghai, China). Full‐length SCAMP1, LMX1A and NLRC5 gene was constructed in pIRES2‐EGFP (GenScript, Piscataway, NJ). miR‐499a‐5p agomir and antagomir, their respective non‐targeting sequence were synthesized from GenePharma. After cells reached 70‐80% confluence in a 24‐well plate, Lipofectamine 3000 and Opti‐MEM I (Life Technologies, Waltham, MA) were used to transfect cells. Then stable transfected cells were selected by G418 (Sigma‐Aldrich, St Louis, MO) screening. The transfection efficacy was detected by qRT‐PCR or Western blot.

### 2.4 RNA isolation and quantitative RT‐PCR (qRT‐PCR)

Total RNA was extracted from the tissues and cells according to Trizol reagent (Life Technologies Corporation, Carlsbad, CA) manufacturer's instructions. SYBR Prime‐Script RT‐PCR Kit (TakaraBio, Japan) was used to detect the mRNA expression levels of SCAMP1, LMX1A, NLRC5 and GAPDH. TaqMan MicroRNA Reverse Transcription kit and TaqMan Universal Master Mix II (Applied Biosystems, Foster City, CA) were used to detect the expression levels of miR‐499a‐5p and U6. After normalizing to the endogenous control GAPDH or U6, the relative expression values were calculated to represent fold change in gene expression by relative quantification (2^−ΔΔCt^ method).

### 2.5 Western blot analysis

Equal amounts of protein samples were separated using 8% or 10% sodiumdodecyl sulphate‐polyacrylamide gel electrophoresis (SDS‐PAGE) and then electrophoretically transferred to a polyvinylidene difluoride membrane (Millipore, Shanghai, China). After blocking 2 hours at room temperature, the membranes were incubated overnight at 4°C with primary antibodies as below: LMX1A (1:1000, abcam, ab31006, EUGENE, USA), NLRC5 (1:1000, abcam, ab105411, EUGENE, USA), β‐catenin, c‐Myc, cyclin D1, MMP‐7 (1:1000, Cell Signaling Technology, Beverly, MA) and GAPDH，β‐actin (1:5000, Proteintech, China). After incubated 2 hours at room temperature with HRP‐conjugated secondary antibodies, protein bands were visualized using ECL (Beyotime) and detected using ECL Detection Systems (Thermo Scientific, Beijing, China). The relative expression was calculated based on the internal control GAPDH or β‐actin.

### 2.6 Cell proliferation assay

U87 and U251 cells were seeded in 96‐well plate (2000 cells per well) and cultured at 37°C for 24 hours. Then 10 μL of Cell Counting Kit‐8 (CCK‐8, Dojin, Japan) solution was added into each well and incubated at 37°C for another 2 hours. The absorbance was measured and recorded at 450 nm.

### 2.7 Cell migration and invasion assays

For migration assay, U87 and U251 cells were resuspended in serum‐free medium at a density of 2 × 10^5^ cells/mL, then 100 μL cell suspension was seeded into the upper chamber of a 24‐well transwell chamber (Costar, Corning, NY), meanwhile 600 μL DMEM/high glucose medium containing 10% FBS was added to the lower chamber. After incubation at 37°C for 24 hours, cells were fixed with methanol and stained with 20% Giemsa solution for 30 minutes at 37°C. Then cell numbers were counted by calculating the average of five random fields under an inverted microscope. For invasion assay, 80 μL of 50 ng/μL Matrigel solution (BD, Franklin Lakes, NJ) was pre‐coated on the transwell membrane.

### 2.8 Apoptosis analysis

Annexin V‐PE/7‐AAD (BD, Biosciences) was employed to detect cell apoptosis. According to the manufacturer's instruction, U87 and U251 cells were collected and washed with cold phosphate‐buffered saline twice, then stained 15 minutes with Annexin V‐PE/7AAD at room temperature. The apoptotic fractions were then analysed using flow cytometry (FACScan, BD Biosciences).

### 2.9 Dual‐luciferase reporter assays

The predicted miR‐499a‐5p binding sequence in SCAMP1 and LMX1A 3' UTR sequence and their corresponding mutant sequence were cloned into pmirGLO Dual‐Luciferase Vector to construct luciferase reporter vector (SCAMP1‐Wt or ‐Mut and LMX1A‐3' UTR‐Wt or ‐Mut; GenePharma). HEK293T cells were seeded into a 96‐well plate and co‐transfected with the indicated vectors and miR‐499a‐5p agomir or its negative control respectively. Dual‐luciferase assay was performed 48 hours after transfection, the relative luciferase activity was normalized to Renilla luciferase activity and calculated by Dual‐Luciferase Reporter Assay System (Promega, Madison, WI).

### 2.10 Human miRNA microarray analysis

The sample preparation, microarray hybridization and miRNAs analysis were performed by Kangchen Bio‐tech (Shanghai, China).

### 2.11 RNA immunoprecipitation

EZ‐Magna RIP kit (Millipore, Billerica, MA) was applied to conduct RNA immunoprecipitation (RIP) assay according to the manufacturer's protocol. Whole cell lysate was incubated with RIP magnetic beads conjugated with anti‐Argonaute2 antibody and normal mouse IgG (Millipore). After incubating with Proteinase K buffer, immunoprecipitated RNA was obtained. Finally, the presence of the binding targets was validated by qRT‐PCR.

### 2.12 Chromatin immunoprecipitation

Simple ChIP Enzymatic Chromatin IP Kit (Cell Signaling Technology) was utilized for Chromatin immunoprecipitation (ChIP) assay according to the manufacturer's protocol. Briefly, cells were crosslinked with EBM‐2 containing 1% formaldehyde for 10 minutes and collected in lysis buffer. Then chromatin was digested by micrococcal nuclease. The immunoprecipitation sample was incubated with 3 μg of anti‐LMX1A antibody or normal rabbit IgG, then gently rotated overnight at 4°C after treating with Protein G Agarose Beads. DNA crosslinks were reversed by 5 mol/L NaCl and proteinase K and were finally purified. DNA was amplified by PCR with respective primers.

### 2.13 Tumour xenografts in nude mice

Stable expression glioma cells for in vivo study was established as following steps. Lentivirus encoding miR‐499a‐5p was generated using pLenti6.3/V5eDEST Gateway Vector Kit (Life Technologies Corporation), while shRNA targeting human SCAMP1 was ligated into the LV3‐CMV‐GFP‐Puro vector (GenePharma). After infection, the stable expressing cells of pre‐miR‐499a‐5p and sh‐SCAMP1 were established. Subsequently, the miR‐499a‐5p lentivirus was transduced in stable expressing cells of sh‐SCAMP1 to produce pre‐miR‐499a‐5p + sh‐SCAMP1 cells. All research methods were conducted strictly in accordance with the protocol of Care and Use of Laboratory Animals, moreover, approvals from the Administrative Panel on Laboratory Animal Care of the Shengjing Hospital also were achieved. Four‐week‐old BALB/C athymic nude mice were obtained from the National Laboratory Animal Center (Beijing, China). The nude mice were divided into four groups (randomized to each group by two performers in a blinded manner, n = 10 per group): Control, sh‐SCAMP1, pre‐miR‐499a‐5p and sh‐SCAMP1 + pre‐miR‐499a‐5p group. For subcutaneous implantation, 3 × 10^5^ cells were subcutaneously injected in the right flanks of the mice. Tumour volume was measured every 4 days using the formula: volume (mm^3^) = length × width^2^/2. The tumour‐bearing mice were executed and tumours were isolated at 40 days after injection. For survival analysis in orthotopic inoculations, 3 × 10^5^ cells were stereotactically implanted into the right striatum of the mice. The number of survived nude mice was recorded and survival analysis was determined using Kaplan‐Meier survival curve.

### 2.14 Statistical analysis

Experimental data are presented as the mean ± standard deviation (SD) from at least three independent experiments. SPSS 22.0 software was used for statistical analysis with the Student's *t* test, one‐way ANOVA, Pearson chi‐square test or Log‐rank test. Differences were considered statistically significant when *P* < 0.05.

## 3 RESULTS

### 3.1 SCAMP1 was up‐regulated in glioma and overexpressed SCAMP1 was correlated with poor prognosis of glioma patients

**Figure 1:**
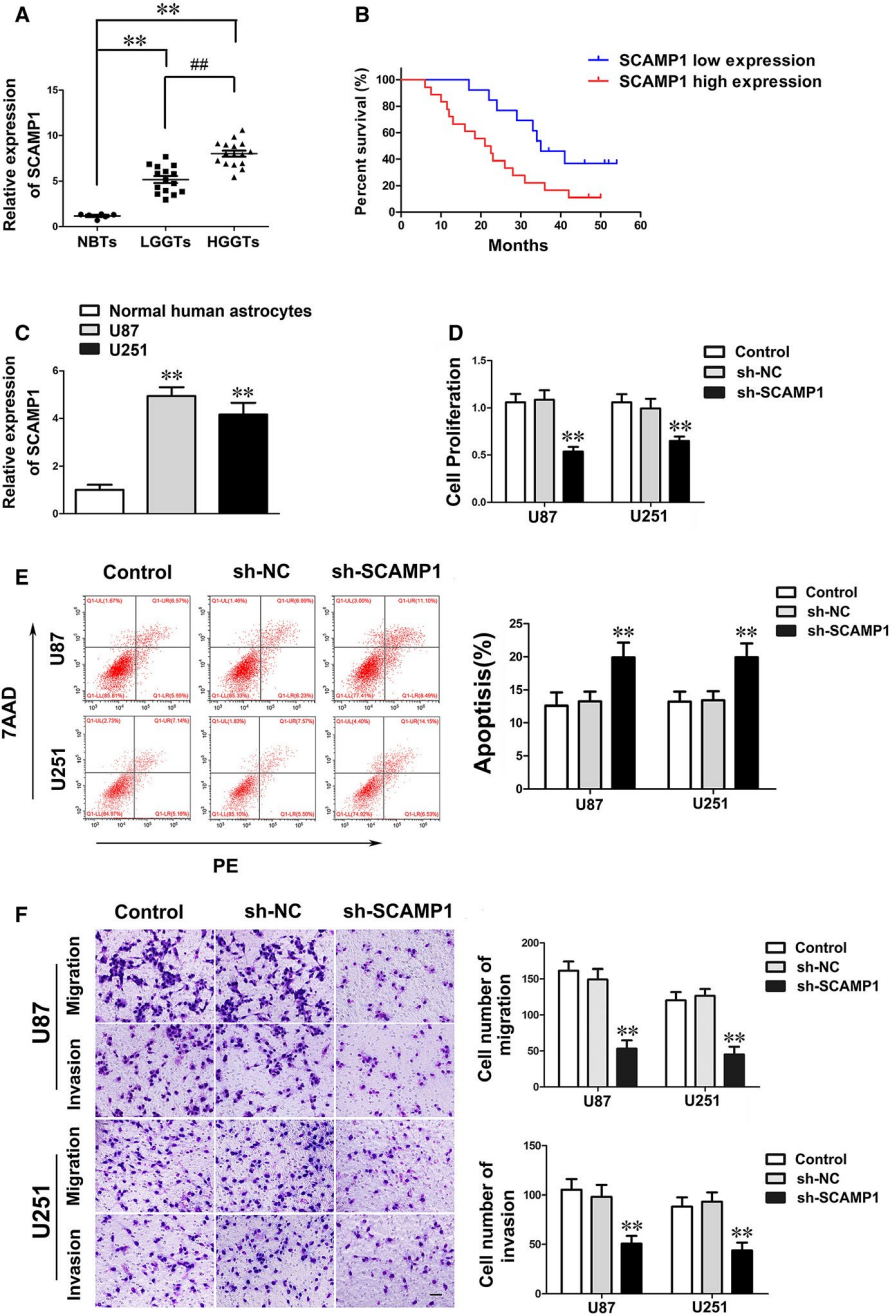
LncRNA SCAMP1 was overexpressed and played oncogenic role in glioma. (A) The expression levels of SCAMP1 were up‐regulated in glioma tissues. Data are presented as the mean ± SD (n = 6, NBTs; n = 15, LGGTs; n = 16, HGGTs). **P < 0.01 vs NBTs group; ^##^ *P* < 0.01 vs LGGTs group. (B) Kaplan‐Meier survival analyses showed that the glioma patients with high expression of SCAMP1 indicated poorer overall survival (log‐rank test, *P* = 0.022). The mean of SCAMP1 expression was used as cut‐off. (C) The SCAMP1 expression levels in NHA and glioma cell lines. Data are presented as the mean ± SD (n = 3, each group). **P < 0.01 vs NHA group. (D) CCK‐8 assay was used to determine the proliferation of U87 and U251 cells treated with SCAMP1 knockdown. (E) The results of flow cytometry analysis in U87 and U251 cells treated with SCAMP1 knockdown. (F) Quantification cell number and representative images of migration and invasion in U87 and U251 cells treated with SCAMP1 knockdown were presented. Scale bars represent 40 μm. Data are given as mean ± SD (n = 3, each group). **P < 0.01 vs sh‐NC group

The expression levels of SCAMP1 in NBTs, glioma tissues, NHA and glioma cell lines were detected by qRT‐PCR. Results showed that SCAMP1 was significantly up‐regulated in glioma tissues and cell lines, moreover, its expression level was positively correlated with the pathological grades of glioma (Figure 1A‐C). Furthermore, Kaplan‐Meier survival analyses and log‐rank test in 31 glioma patients suggested that higher SCAMP1 expression indicated poorer overall survival (Figure 1B). The correlation analyses between SCAMP1 expression levels and clinicopathological features of 31 glioma patients are also displayed in Table S1. For deeply investigating the functions of SCAMP1 in glioma, we up‐regulated and down‐regulated the expression of SCAMP1 in glioma cells, then the effects on malignant biological behaviours were detected by CCK‐8 assay, flow cytometry analysis and transwell assay. As shown in Figure 1D, knockdown of SCAMP1 significantly inhibited the proliferation ability while facilitated apoptosis of glioma cells compared with the sh‐NC group (Figure 1E). Moreover, reduction in SCAMP1 exhibited weaker cell migration and invasion ability than sh‐NC group (Figure 1F). On the contrary, up‐regulation of SCAMP1 remarkably enhanced the proliferation, migration and invasion ability, meanwhile decreased apoptosis of glioma cells (Figure S2A‐C). Data above suggested that SCAMP1 acted as an oncogene in glioma cells.

### 3.2 miR‐499a‐5p was down‐regulated and manifested a tumour suppressor in glioma

**Figure 2:**
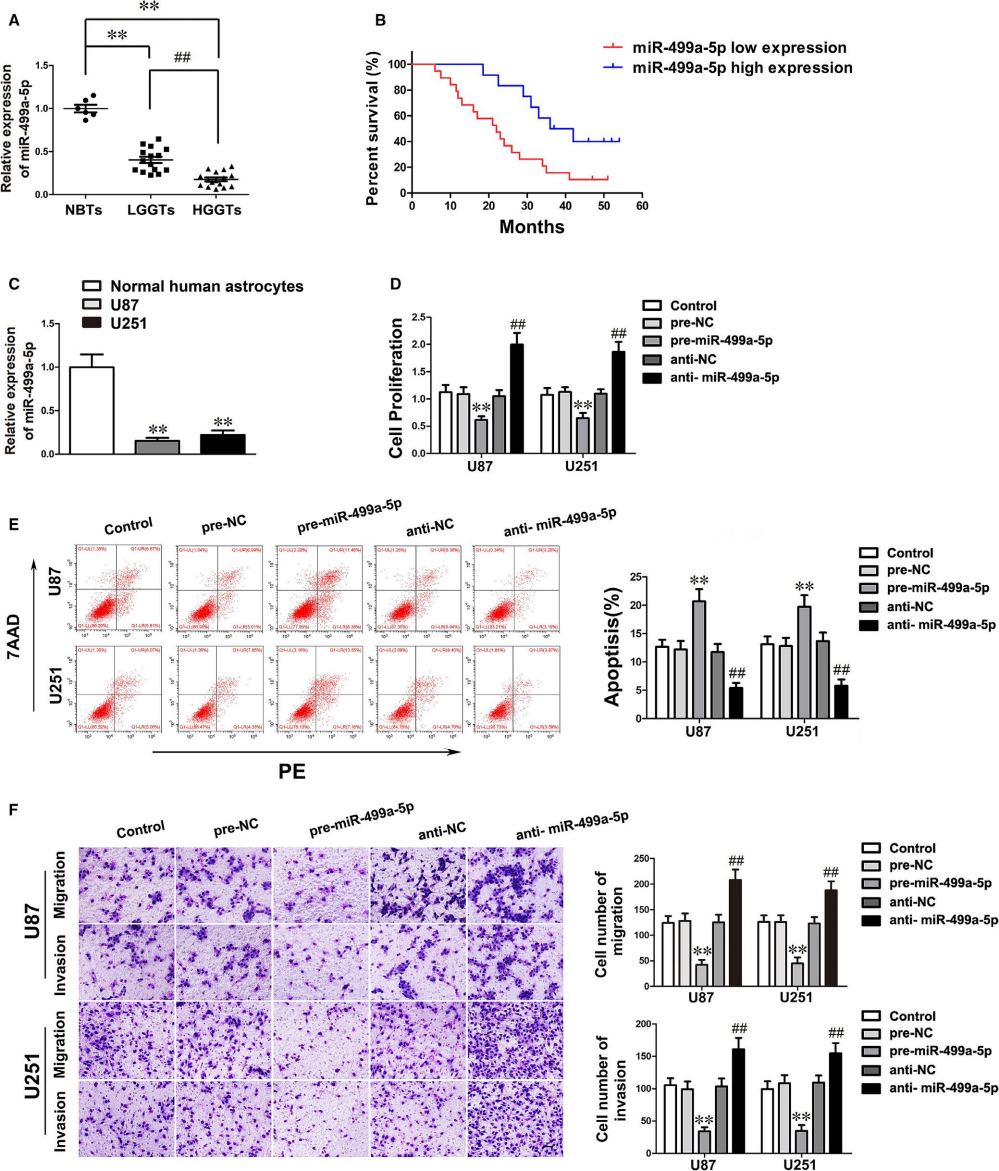
miR‐499a‐5p was low expressed and manifested a tumour suppressor in glioma. (A) The expression levels of miR‐499a‐5p were down‐regulated in glioma tissues. Data are presented as the mean ± SD (n = 6, NBTs; n = 15, LGGTs; n = 16, HGGTs). **P < 0.01 vs NBTs group; ^##^ *P* < 0.01 vs LGGTs group. (B) Glioma patients with low expression of miR‐499a‐5p exhibited worse overall survival (log‐rank test, *P* = 0.011). The mean of miR‐499a‐5p expression was used as cut‐off. (C) The expression levels of miR‐499a‐5p in NHA, U87 and U251 cells. Data are presented as the mean ± SD (n = 3, each group). **P < 0.01 vs NHA group. (D‐F) The effects of miR‐499a‐5p on malignant biological behaviours of U87 and U251 cells were presented by CCK‐8 assay, flow cytometry analysis and transwell assay. Scale bars in transwell assay represent 40 μm. Data are given as mean ± SD (n = 3, each group). **P < 0.01 vs pre‐NC group; ^##^ *P* < 0.01 vs anti‐NC group

miRNAs microarray analysis was employed to identify that miR‐499a‐5p was remarkably up‐regulated in glioma cells when SCAMP1 knockdown, implying miR‐499a‐5p may participate in SCAMP1‐induced modulation on glioma cells (Figure S1). Subsequently, as shown in Figure 2A‐C, miR‐499a‐5p expression was proven to be significantly down‐regulated in glioma tissues, U87 and U251 cell lines compared with NBTs and NHA respectively. Similarly, the expression level of miR‐499a‐5p was negatively correlated with the progression of glioma pathological grade. Moreover, Kaplan‐Meier survival analyses demonstrated that lower miR‐499a‐5p expression levels led to poorer prognosis of glioma patients and were associated with advanced pathological grade in 31 glioma patients (Figure 2B and Table S2). To assess the functions of miR‐499a‐5p in glioma, U87 and U251 cells were transfected with miR‐499a‐5p‐agomir or antagomir. As shown in Figure 2D‐F, up‐regulation of miR‐499a‐5p significantly decreased the proliferation, migration and invasion ability, meanwhile enhanced apoptosis of glioma cells compared with the pre‐NC group. These evidence above inferred that miR‐499a‐5p exerted tumour‐suppressive functions in glioma cells.

### 3.3 miR‐499a‐5p targeted SCAMP1 and its expression was negatively regulated by SCAMP1

**Figure 3:**
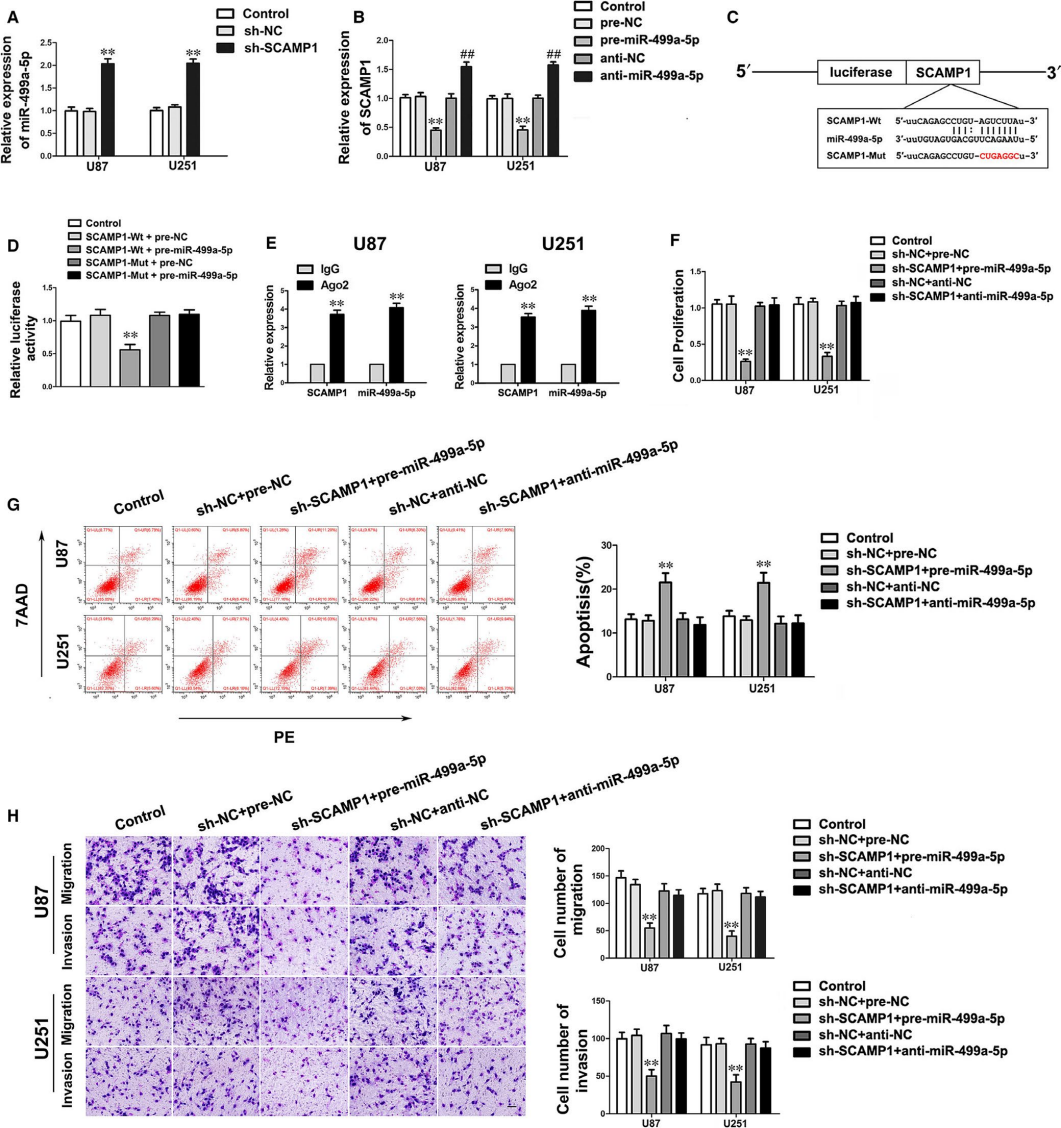
SCAMP1 was a target of miR‐499a‐5p, moreover, miR‐499a‐5p could mediate the effects of SCAMP1 knockdown on glioma cells. (A) The expression of miR‐499a‐5p after SCAMP1 knockdown in glioma cells. Data are presented as the mean ± SD (n = 3, each group). **P < 0.01 vs sh‐NC group. (B) The expression of SCAMP1 in U87 and U251 cells transfected with miR‐499a‐5p agomir and antagomir. Data are presented as the mean ± SD (n = 3, each group). **P < 0.01 vs pre‐NC group; ^##^ *P* < 0.01 vs anti‐NC group. (C‐D) The predicted miR‐499a‐5p binding site in the SCAMP1 sequence (SCAMP1‐Wt) and the designed mutant sequence of miR‐499a‐5p binding site (SCAMP1‐Mut) are indicated. Relative luciferase activity was detected after cells were co‐transfected with pre‐miR‐499a‐5p and SCAMP1‐Wt or SCAMP1‐Mut. Data are presented as the mean ± SD (n = 3, each group). **P < 0.01 vs SCAMP1‐Wt + pre‐NC group. (E) miR‐499a‐5p was identified in the SCAMP1‐RISC complex. SCAMP1 and miR‐499a‐5p enrichment were measured using qRT‐PCR. Data represent mean ± SD (n = 3, each group). **P < 0.01 vs anti‐IgG group. (F‐H) The malignant biological behaviours of U87 and U251 cells co‐transfected with sh‐SCAMP1 and miR‐499a‐5p agomir or antagomir were measured by CCK‐8 assay, flow cytometry analysis and transwell assay. Scale bars in transwell assay represent 40 μm. Data are presented as the mean ± SD (n = 3, each group). **P < 0.01 vs sh‐NC + pre‐NC group

Using bioinformatics database (Starbase), SCAMP1 was identified as a potential target of miR‐499a‐5p. To clarify whether SCAMP1 could bind to miR‐499a‐5p through the putative binding site, the expression of miR‐499a‐5p in U87 and U251 cells transfected with sh‐SCAMP1 was firstly detected. Results showed that the miR‐499a‐5p expression was significantly up‐regulated after SCAMP1 knockdown (Figure 3A). Conversely, the SCAMP1 expression in glioma cells transfected with miR‐499a‐5p agomir was remarkably decreased (Figure 3B). Dual‐luciferase assay was further quantified the interaction between SCAMP1 and miR‐499a‐5p. As shown in Figure 3C‐D, the luciferase activity in SCAMP1‐Wt + pre‐miR‐499a‐5p group was considerably attenuated when compared with that in SCAMP1‐Wt + pre‐NC group. In addition, RIP assay showed that the expressions of SCAMP1 and miR‐499a‐5p were both increased in the anti‐Ago2 group compared with that in anti‐normal group, suggesting SCAMP1 and miR‐499a‐5p were in an RNA‐induced silencing complex (RISC) (Figure 3E). Therefore, results above indicated that there was a reciprocal repression feedback loop between SCAMP1 and miR‐499a‐5p.

### 3.4 miR‐499a‐5p mediated the tumour‐suppressive effects of SCAMP1 knock‐down on glioma cells

To investigate whether miR‐499a‐5p could regulate the effects of SCAMP1 inhibition on glioma cells, U87 and U251 cells were co‐transfected with sh‐SCAMP1 and miR‐499a‐5p agomir or antagomir. The results of CCK‐8 assay, transwell assay and flow cytometry analysis indicated that sh‐SCAMP1 + pre‐miR‐499a‐5p group remarkably led to a decrease in proliferation, migration and invasion of glioma cells, while facilitated cell apoptosis compared with the sh‐NC + pre‐NC group (Figure 3F‐H). Moreover, down‐regulation of miR‐499a‐5p obviously rescued the inhibitory effects of SCAMP1 knockdown on biological behaviours of glioma cells (Figure 3F‐H).

### 3.5 Knockdown of LMX1A restrained cell proliferation, migration and invasion of glioma cells while promoting apoptosis

**Figure 4:**
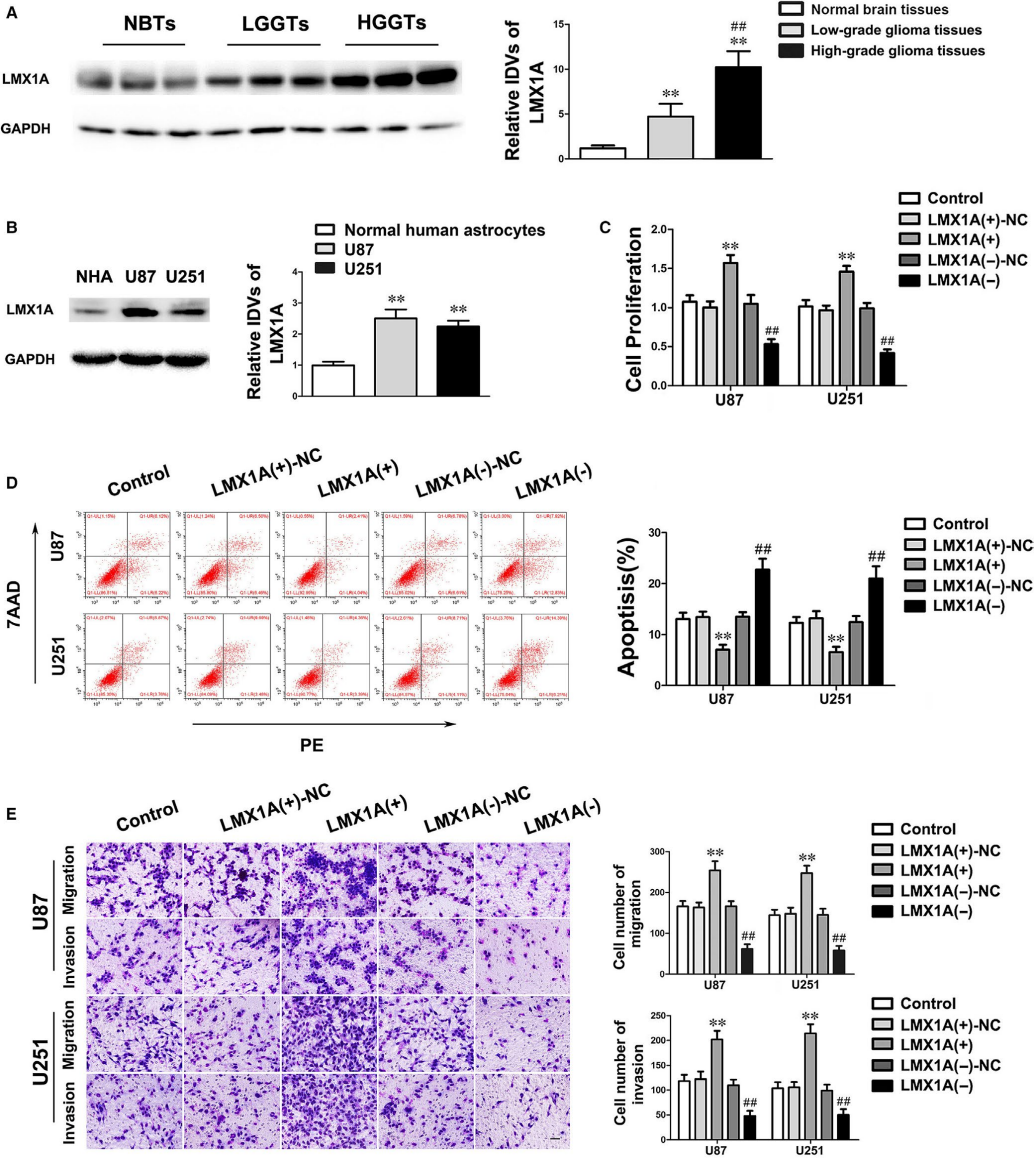
LMX1A was up‐regulated and exerted cancerogenic functions in glioma. (A) The protein expression levels of LMX1A in NBTs, LGGTs and HGGTs were detected by Western blot. Data are presented as the mean ± SD (n = 6, NBTs; n = 15, LGGTs; n = 16, HGGTs). **P < 0.01 vs NBTs group; ^##^ *P* < 0.01 vs LGGTs group. (B) The endogenous expression levels of LMX1A in NHA and glioma cell lines U87 and U251. (C‐E) The effects of LMX1A on malignant biological behaviours of U87 and U251 cells were presented by CCK‐8 assay, flow cytometry analysis and transwell assay. Scale bars in transwell assay represent 40 μm. Data are given as mean ± SD (n = 3, each group). **P < 0.01 vs LMX1A(+)‐NC group; ^##^ *P* < 0.01 vs LMX1A(−)‐NC group

Bioinformatics database (TargetScan) was used to identify that LMX1A was a potential downstream target of miR‐499a‐5p. To confirm our hypothesis, the expression levels of LMX1A in glioma tissues and cells were detected. Western blot assay showed that LMX1A was up‐regulated in glioma tissues and cell lines compared with NBTs and NHA, moreover, the expression level was positively correlated with pathological grades of glioma (Figure 4A‐B). Remarkably, knockdown of LMX1A significantly restrained the biological behaviours of glioma cells compared with LMX1A(‐)‐NC group (Figure 4C‐E). These evidence indicated that LMX1A played a cancerogenic role in malignant progression of glioma cells.

### 3.6 miR‐499a‐5p directly targeted LMX1A‐3′‐UTR and suppressed LMX1A‐induced malignant biological behaviour in glioma cells

**Figure 5:**
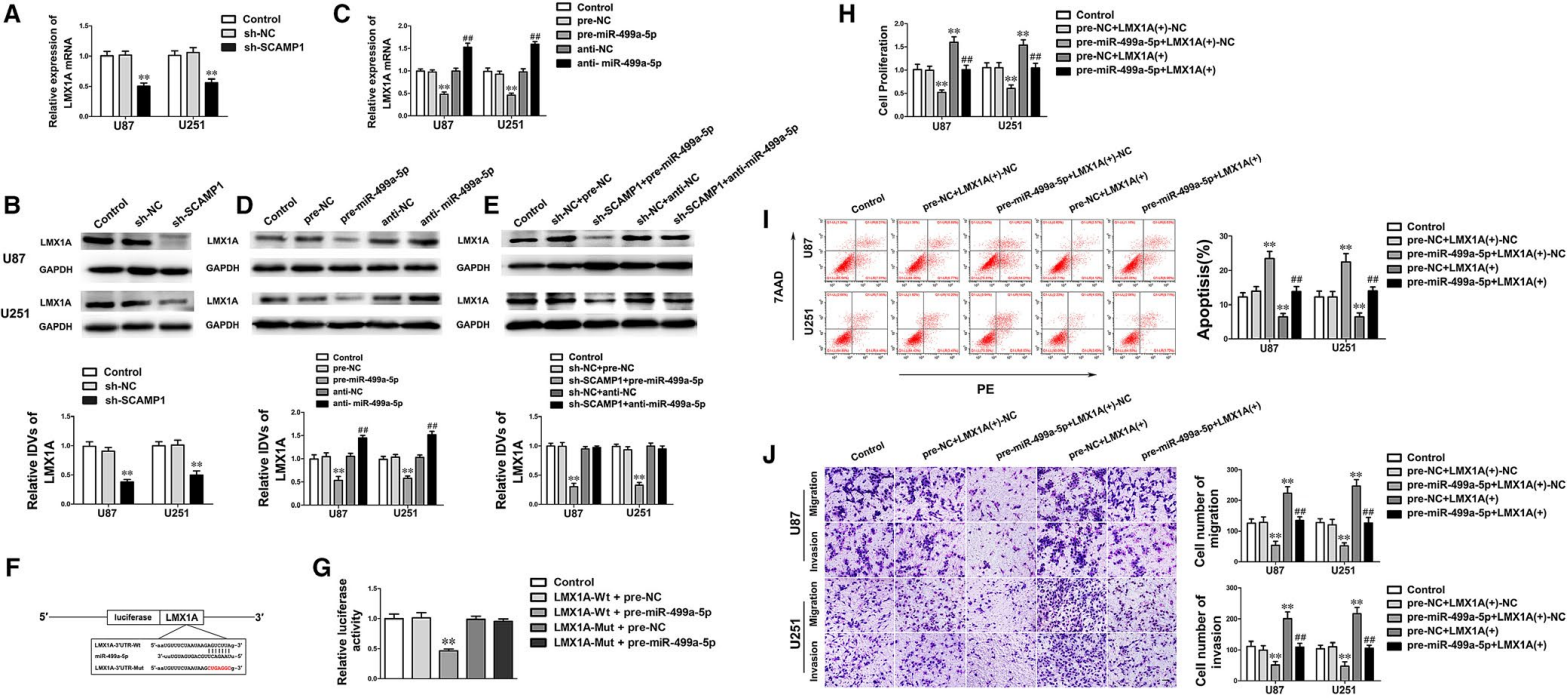
LMX1A was a target of miR‐499a‐5p and rescued the tumour inhibitory effects of miR‐499a‐5p. (A‐B) qRT‐PCR and Western blot assay were used to detect the LMX1A expression after SCAMP1 knockdown. (C‐D) qRT‐PCR and Western blot assay were used to examine the LMX1A expression of U87 and U251 cells after altered miR‐499a‐5p expression. (E) Western blot assay was used to detect the LMX1A expression of U87 and U251 cells co‐transfected with SCAMP1 and miR‐499a‐5p. (F) The potential miR‐499a‐5p binding sites in LMX1A‐3′‐UTR and the designed mutant sequence are indicated. (G) Relative luciferase activity was detected after cells were co‐transfected with pre‐miR‐499a‐5p and LMX1A‐Wt or LMX1A‐Mut. Data are presented as the mean ± SD (n = 3, each group). **P < 0.01 vs LMX1A‐Wt + pre‐NC group. (H‐J) The malignant biological behaviours of U87 and U251 cells co‐transfected with miR‐499a‐5p and LMX1A were detected by CCK‐8 assay, flow cytometry analysis and transwell assay. Scale bars in transwell assay represent 40 μm. Data are presented as the mean ± SD (n = 3, each group). **P < 0.01 vs pre‐NC + LMX1A (+)‐NC group, ^##^ *P* < 0.01 vs pre‐miR‐499a‐5p + LMX1A (+)‐NC group

The previous results uncovered that LMX1A exerted cancerogenic functions in glioma cells, however whether LMX1A was involved in SCAMP1 and miR‐499a‐5p regulatory malignant progression of glioma cells remains blurry. Bioinformatics database (Targetscan) speculated that miR‐499a‐5p may target LMX1A‐3′‐UTR. Hence the LMX1A expression of glioma cells altered SCAMP1 and miR‐499a‐5p expression was firstly detected. Results showed that SCAMP1 knockdown or miR‐499a‐5p overexpression obviously inhibited LMX1A mRNA and protein expression (Figure 5A‐D). Furthermore, the expression of LMX1A in glioma cells co‐transfected with sh‐SCAMP1 and miR‐499a‐5p agomir or antagomir was examined. Results showed that down‐regulation of miR‐499a‐5p expression dramatically reversed the suppressive effect on LMX1A by SCAMP1 knockdown in glioma cells (Figure 5E). To deeply clarify the interaction between miR‐499a‐5p and LMX1A, dual‐luciferase assay was carried out (Figure 5F‐G). The results above demonstrated that miR‐499a‐5p directly targeted the specific binding sequence in the LMX1A‐3′‐UTR.

To further explore whether LMX1A could mediate the tumour‐suppressive effects of miR‐499a‐5p, down‐regulated LMX1A by pre‐miR‐499a‐5p was reversed prior to the assessment of the malignant biological behaviours in glioma cells. The results showed that overexpressed LMX1A significantly rescued the inhibitory effect of overexpressed miR‐499a‐5p on cell proliferation, migration and invasion, moreover diminished cell apoptotic percentage (Figure 5H‐J). These findings above indicated that miR‐499a‐5p suppressed the malignant progression of glioma cells via down‐regulating LMX1A expression.

### 3.7 NLRC5 was involved in LMX1A‐mediated modulation on malignant progression of glioma cells and exerted an oncogenic role via activating the Wnt/β‐catenin signalling pathway

**Figure 6:**
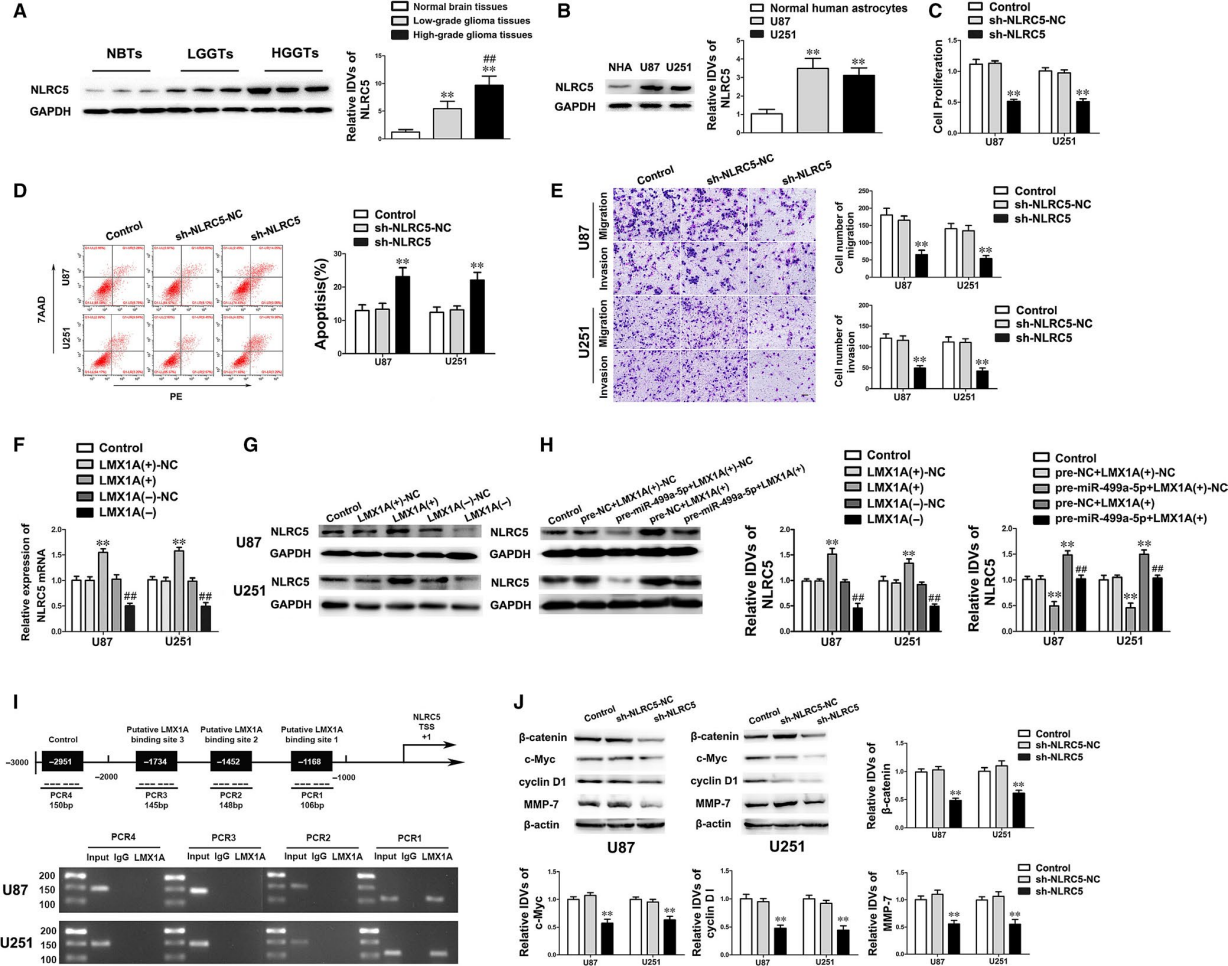
NLRC5 was a downstream target of LMX1A and conducted oncogenic effects on glioma cells by activating the Wnt/β‐catenin signalling pathway. (A) The expression levels of NLRC5 in NBTs and glioma tissues were detected by Western blot. Data are presented as the mean ± SD (n = 6, NBTs; n = 15, LGGTs; n = 16, HGGTs). **P < 0.01 vs NBTs group; ^##^ *P* < 0.01 vs LGGTs group. (B) The expression levels of NLRC5 in NHA and glioma cell lines. Data are presented as the mean ± SD (n = 3, each group). **P < 0.01 vs NHA group. (C‐E) The effects of NLRC5 on malignant biological behaviours of U87 and U251 cells were investigated by CCK‐8 assay, flow cytometry analysis and transwell assay. Scale bars in transwell assay represent 40 μm. Data are presented as mean ± SD (n = 3, each group). **P < 0.01 vs sh‐NLRC5‐NC group. (F‐G) The mRNA and protein expression levels of NLRC5 were measured by qRT‐PCR and Western blot. Data are presented as the mean ± SD (n = 3, each group). **P < 0.01 vs LMX1A(+)‐NC group; ^##^ *P* < 0.01 vs LMX1A(−)‐NC group. (H) Western blot assay showed that the NLRC5 expression was regulated by miR‐499a‐5p and LMX1A. Data are presented as the mean ± SD (n = 3, each group). **P < 0.01 vs pre‐NC + LMX1A(+)‐NC group, ^##^ *P* < 0.01 vs pre‐miR‐499a‐5p + LMX1A(+)‐NC group. (I) ChIP assay demonstrated that LMX1A directly bound to the promoter of NLRC5 in U87 and U251 cells. As schematic representation displayed, the putative LMX1A binding site located at the NLRC5 promoter region 1168 bp upstream of TSS. Immunoprecipitated DNA was amplified by PCR. Normal rabbit IgG was used as a negative control. (J) Western blot assay showed the expression levels of β‐catenin, c‐Myc, cyclin D1, MMP‐7 in U87 and U251 cells treated with NLRC5 knockdown. Data are presented as the mean ± SD (n = 3, each group). **P < 0.01 vs sh‐NLRC5‐NC group

NLRC5 was inferred as a downstream gene of LMX1A by the bioinformatics databases (DBTSS HOME and JASPAR). To ascertain our hypothesis, the expression levels of NLRC5 in glioma tissues and cells were firstly detected. As shown in Figure 6A‐B, NLRC5 expression was significantly up‐regulated in glioma tissues and glioma cell lines compared with NBTs and NHA and the expression level was positively correlated with pathological grades of glioma. In addition, NLRC5 knockdown robustly inhibited the ability of cell proliferation, migration and invasion, while augmented cell apoptosis versus sh‐NLRC5‐NC group (Figure 6C‐E). Conversely, up‐regulation of NLRC5 expression remarkably enhanced the malignant biological behaviours of glioma cells (Figure S3A‐C). These evidence above suggested that NLRC5 functioned oncogenic role in glioma cells.

In the further studies, the expression of NLRC5 in glioma cells treated with altering miR‐499a‐5p and LMX1A expression was inspected. As shown in Figure 6F‐G, LMX1A knockdown obviously reduced NLRC5 expression. Furthermore, overexpression of LMX1A rescued the tumour‐suppressive impacts of overexpressed miR‐499a‐5p on glioma cells via up‐regulating NLRC5 expression (Figure 6H). Then ChIP assay deeply affirmed the interaction between NLRC5 and LMX1A. The promoter sequence of NLRC5 was established according to the bioinformatics database (DBTSS HOME). The potential binding region in the promoter was identified when scanning the DNA sequence in the −2000 to +200 bp region of the transcription start site (TSS). As a negative control, PCR was conducted to amply the region 1000 bp upstream of the putative NLRC5 binding sequence. As ChIP results corroborated, there was a direct interaction between LMX1A and NLRC5, while no association was found between LMX1A and the control region (Figure 6I). Overall, evidence above revealed that miR‐499a‐5p suppressed the expression of NLRC5 by reducing LMX1A in glioma cells.

Accumulating reports suggested that deregulation of Wnt/β‐catenin signalling pathway is considered as an important marker in various human tumours.^23^ Nevertheless, whether Wnt/β‐catenin signalling pathway was involved in NLRC5‐induced malignant progression of glioma cells remains elusive. To deeper reveal the molecular mechanism of the NLRC5 oncogenic roles, the activity of Wnt/β‐catenin signalling pathway was examined. As shown in Figure 6J, the expression of β‐catenin, c‐Myc, cyclin D1 and MMP‐7 was significantly down‐regulated after NLRC5 was knockdown. Furthermore, overexpressed NLRC5 remarkably up‐regulated the activity of Wnt/β‐catenin signalling pathway (Figure S3D). Consequently, data above determined that NLRC5 accelerated malignant progression of glioma cells via enhancing the Wnt/β‐catenin signalling pathway.

### 3.8 Down‐regulation of SCAMP1 combined with overexpression of miR‐499a‐5p inhibited tumour xenograft growth while exhibited higher survival ability

**Figure 7:**
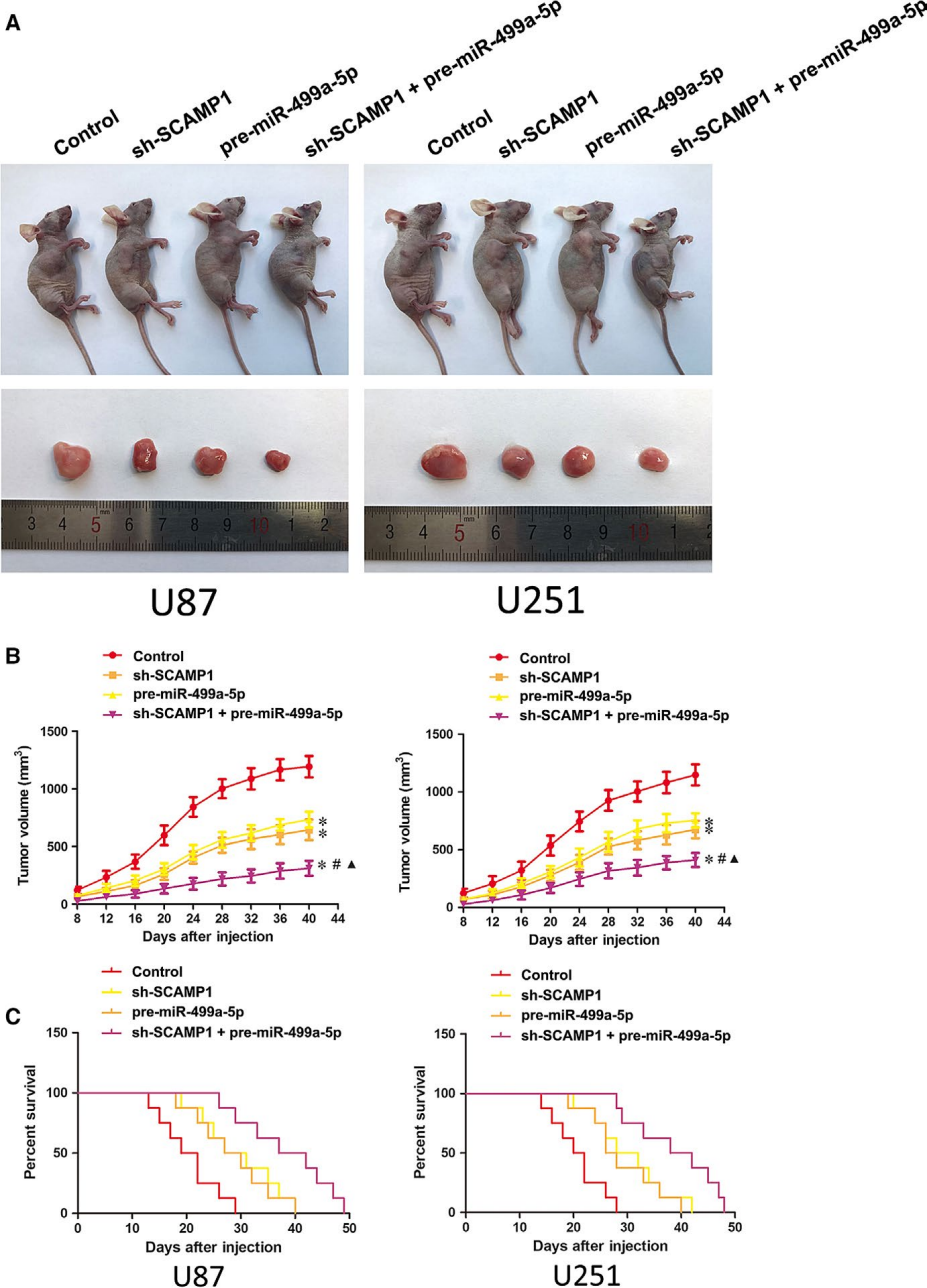
In vivo study. (A) The nude mice and the sample tumours from respective groups were shown. (B) Tumour xenograft growth curves in subcutaneous implantation assay were shown. Tumour volume was calculated every 4 days after injection and the tumour was resected after 40 days. Data represent mean ± SD (n = 10, each group). *P < 0.05 vs control group, ^#^ *P* < 0.05 vs sh‐SCAMP1 group, ^▲^ *P* < 0.05 vs pre‐miR‐499a‐5p group. (C) Survival curves from representative nude mice injected into the right striatum in orthotopic inoculations assay were shown (n = 10, each group). *P* < 0.05 (sh‐SCAMP1, pre‐miR‐499a‐5p and sh‐SCAMP1 + pre‐miR‐499a‐5p groups vs control group)

In vivo study further ascertained the above findings. The results showed that the tumour volumes of sh‐SCAMP1, pre‐miR‐499a‐5p and sh‐SCAMP1 + pre‐miR‐499a‐5p group were smaller than the control group. Additionally, down‐regulation of SCAMP1 combined with overexpression of miR‐499a‐5p presented the smallest tumour volume among all groups (Figure 7A‐B). As shown in Figure 7C, the survival analysis in orthotopic inoculations assay manifested that the mice in the sh‐SCAMP1, pre‐miR‐499a‐5p and sh‐SCAMP1 + pre‐miR‐499a‐5p groups obtained longer survival time compared with control group, moreover the mice treated with sh‐SCAMP1 + pre‐miR‐499a‐5p had the longest survival time.

## 4 DISCUSSION

In this study, we confirmed that SCAMP1 was overexpressed in glioma tissues and cell lines. In addition, SCAMP1 knockdown significantly inhibited the malignant biological behaviours of glioma cells. On the contrary, the miR‐499a‐5p expression was down‐regulated and functioned as a tumour suppressor in glioma cells. Furthermore, we affirmed that miR‐499a‐5p expression was negatively regulated by the miRNA sponges role of SCAMP1. LMX1A and NLRC5 were highly expressed in glioma tissues and cell lines, knockdown of LMX1A or NLRC5 obviously impeded the malignant progression of glioma cells. Moreover, the LMX1A expression was inhibited by miR‐499a‐5p via directly targeting the LMX1A 3′‐UTR, meanwhile the NLRC5 expression could be stimulated by LMX1A through binding to its promoter region in a sequence‐dependent manner. Consequently, the molecular mechanism revealed that knockdown of SCAMP1 increased miR‐499a‐5p expression, which hindered the expression of LMX1A and NLRC5, further led to carcinostatic effects on glioma cells by repressing the activity of Wnt/β‐catenin signalling pathway. Strikingly, SCAMP1 knockdown combined with miR‐499a‐5p overexpression predominantly reduced xenograft tumour growth and elongated the nude mice survival time.

Recently, lncRNAs have been proved to play an important regulatory role on the malignant progression of copious human tumours.^24^, ^25^, ^26^ In particular, different lncRNAs exhibit distinct gene expression patterns in glioma, such as CRNDE and Linc00152, promoting malignant progression of glioma, while Gas5 exerts tumour‐suppressive functions in glioma cells.^27^, ^28^, ^29^ In previous studies, SCAMP1 was reported as a key functional protein in various tumours, for example, up‐regulated SCAMP1 promoted cell migration and invasion in human pancreatic and gallbladder cancer cells.^10^ However, there is no testimony concerned with the role of SCAMP1 as a lncRNA in human tumours. Hence, the biological effects of SCAMP1 as a lncRNA on glioma should be penetratingly investigated. Our data showed LncRNA SCAMP1 was up‐regulated in glioma tissues and cell lines and inhibition of SCAMP1 expression significantly suppressed the cell proliferation, migration and invasion, moreover, facilitated apoptosis of glioma cells. For the first time, these results revealed that SCAMP1 acted as a lncRNA conducted carcinogenic functions in glioma cells, whereas the underlying mechanisms need to be further clarified.

Increasing evidence have proven the cross‐regulation between lncRNAs and miRNAs.^30^, ^31^ LncRNAs may act as competing endogenous RNA (ceRNA) to modulate the expression and function of miRNAs, which also known as miRNA sponges.^32^, ^33^ To ascertain the specific carcinogenic mechanism of SCAMP1 in glioma, bioinformatics database (Starbase) was employed to identify miR‐499a‐5p as an emerging target of SCAMP1. The results of dual‐luciferase and RIP assay confirmed this conjecture that miR‐499a‐5p bound to SCAMP1 and they were involved in an RISC complex. Further experiments showed that miR‐499a‐5p expression was negatively correlated with SCAMP1 expression and SCAMP1 knockdown obviously up‐regulated miR‐499a‐5p in glioma. Meanwhile, overexpression of miR‐499a‐5p reversed the tumour inhibitory effects induced by SCAMP1 knockdown. These results suggested that SCAMP1 and miR‐499a‐5p forming a potential reciprocal repression feedback loop. Similarly, this interaction between lncRNAs and miRNAs was also affirmed by other studies in glioma cells. OIP5‐AS1 and miR‐367‐3p could reciprocally regulate the expression of each other, affect the biological behaviours of glioma cells together.^34^ SOX2OT inhibited the malignant progression of glioma stem cells via up‐regulating the expression of miR‐194‐5p and miR‐122.^35^

miRNAs are considered as an indispensable link of well‐known oncogenic and tumour suppressor networks and they participate in the occurrence and development of various human tumours.^36^, ^37^, ^38^ miR‐499a‐5p conducted as a tumour suppressor in osteosarcoma cells and suppressed cell proliferation and differentiation by targeting protein phosphatase 1D.^14^ It also exerted tumour‐suppressive functions in oesophageal carcinoma cells by enhancing the cisplatin sensitivity.^15^ In order to uncover the role of miR‐499a‐5p in glioma, the expression levels of miR‐499a‐5p in glioma tissues and cell lines were firstly examined. The results showed that miR‐499a‐5p expression was significantly down‐regulated and restoration of miR‐499a‐5p dramatically restrained the malignant evolution of glioma cells. Similarly, overexpression of miR‐499a‐5p inhibited cell proliferation and metastasis, acted as a tumour suppressor in non‐small cell lung cancer.^39^ Moreover, in vivo studies confirmed that the nude mice treated with SCAMP1 knockdown combining with overexpressed miR‐499a‐5p manifested the smallest tumour volume and longest survival time. In brief, SCAMP1 played an oncogenic role in glioma cells via repressing miR‐499a‐5p expression, however, the deeper mechanisms of miR‐499a‐5p‐induced tumour‐suppressive effects were still unclear.

miRNAs could post‐transcriptionally regulate the expression of downstream gene via binding its 3′‐UTR. miR‐139 regulated malignant behaviours of glioma cells through directly targeting RUNX1 3′‐UTR.^40^ miR‐370 was also reported that exerted tumour‐suppressive function in glioma cells by directly binding to CCNE2 3′‐UTR.^41^ In our study, using bioinformatics database (TargetScan) combined with dual‐luciferase assay certified that LMX1A was directly targeted by miR‐499a‐5p. Then we discovered that LMX1A expression was negatively modulated by miR‐499a‐5p through binding to LMX1A 3′‐UTR. In addition, our research showed LMX1A was overexpressed in glioma tissues and cell lines, meanwhile, up‐regulation of LMX1A significantly enhanced the proliferation, migration and invasion as well as suppressed apoptosis, implying that LMX1A exerts oncogenic effects on glioma cells. In earlier studies, LMX1A expression was found to be up‐regulated in glioma and showed positive correlation with histologic grade and clinical stage, which was consistent with our finding.^42^ More importantly, results showed that overexpressed LMX1A obviously rescued the miR‐499a‐5p‐induced tumour‐suppressive effects on glioma cells, expositing that miR‐499a‐5p impeded the malignant progression of glioma cells via reducing LMX1A expression.

NLRC5 belongs to NLR family and plays a physiologically important role in the maintenance of immune homoeostasis.^43^ Recently, the oncogenic role of NLRC5 in human tumours immensely attracts people's attention.^44^, ^45^ In our research, the expression levels of NLRC5 were found that significantly elevated in glioma tissues and cell lines. In addition, NLRC5 knockdown remarkably restrained the biological behaviours of glioma cells, which demonstrating that NLRC5 acts as a carcinogen in glioma cells. In the earlier studies, NLRC5 was also affirmed highly expressed in hepatocellular carcinoma cells and promoted cell proliferation.^44^ What's more, high expression of NLRC5 was correlated with poor survival of non‐small cell lung cancer patients.^45^ These studies further supported our findings.

As mentioned above, using bioinformatics database (DBTSS HOME and JASPAR), we discovered that the promoter region of NLRC5 contained the potential binding sequence of LMX1A, indicating NLRC5 might be a downstream target of LMX1A. Our further research showed overexpression of miR‐499a‐5p significantly decreased NLRC5 expression, further inhibited the biological behaviours of glioma cells. Whereas overexpressed LMX1A produced contrary effects. Additionally, overexpression of LMX1A partly rescued the miR‐499a‐5p‐induced tumour‐suppressive effects through elevating the NLRC5 expression. Furthermore, ChIP assay also demonstrated that LMX1A could activate NLRC5 transcription through directly binding to the specific sequence of NLRC5 promoter. Overall, miR‐499a‐5p could negatively regulate the expression of LMX1A, which directly bound to NLRC5 promoter and modulated its expression, then suppressed the biological behaviours of glioma cells.

**Figure 8:**
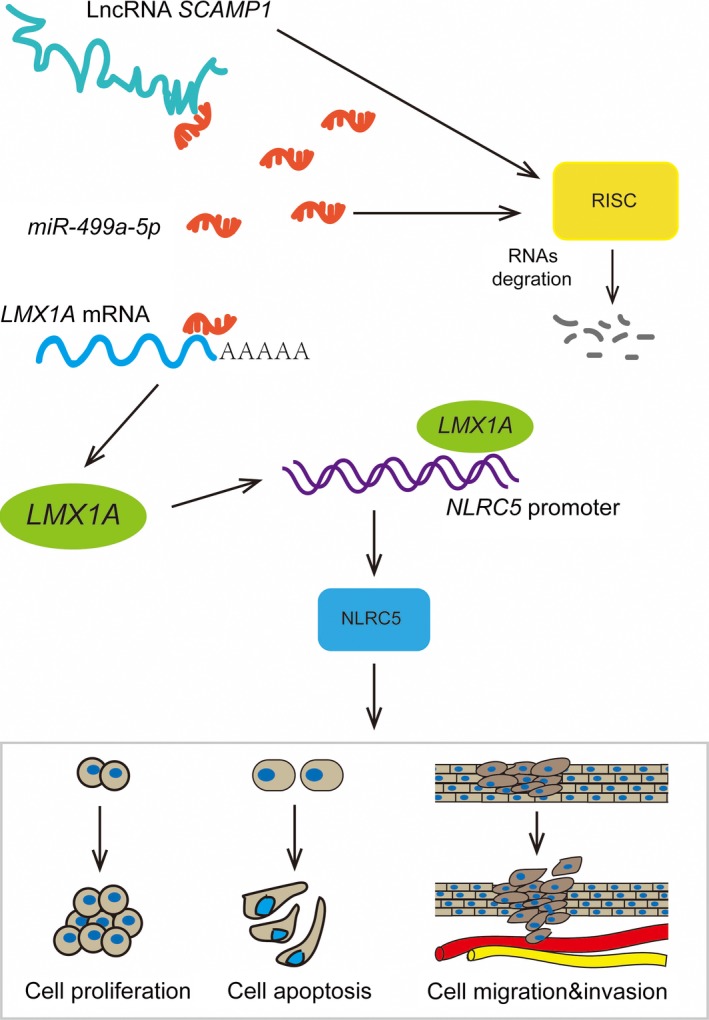
The schematic diagram underlying the mechanism of SCAMP1/miR‐499a‐5p/LMX1A/NLRC5 axis in glioma cells

Emerging evidence have shown that aberrant activation of the Wnt/β‐catenin signalling pathway plays a crucial modulatory role in the initiation, progression and metastasis of various human tumours, such as colorectal cancer, breast cancer and particularly glioma.^46^, ^47^, ^48^, ^49^, ^50^ Some earlier studies certified that the Wnt/β‐catenin signalling pathway was involved in plenty of malignant biological processes of glioma. For instance, Wnt/β‐catenin signalling pathway was activated by NEAT1 and then promoted glioma cells growth and invasion.^51^ Moreover, inhibition of the Wnt/β‐catenin signalling pathway by miR‐96 knockdown suppressed the proliferation and colony formation of glioma cells.^52^ In previous research, we found the Wnt/β‐catenin signalling pathway was stimulated by NLRC5 and played oncogenic role in hepatocellular carcinoma^22^. However, whether Wnt/β‐catenin signalling pathway participated in NLRC5‐induced malignant behaviours in glioma cells needs to be explored. Our studies revealed that reduction in NLRC5 abundance significantly repressed the activity of Wnt/β‐catenin signalling pathway in glioma cells. Therefore, data above clarified that LMX1A knockdown obviously attenuated the activity of Wnt/β‐catenin signalling pathway by down‐regulating NLRC5 expression and restrained malignant behaviours of glioma cells. The underlying molecular mechanism of the tumour‐suppressive functions elicited by SCAMP1 knockdown is schematically presented in Figure 8.

In summary, our study revealed that LncRNA SCAMP1 plays an oncogenic role while miR‐499a‐5p exerts tumour‐suppressive functions in glioma cells. SCAMP1 could increase LMX1A by negative regulating miR‐499a‐5p expression in glioma cells. LMX1A transcriptionally activates the NLRC5 expression, which promotes the malignant biological behaviours of glioma cells through stimulating Wnt/β‐catenin signalling pathway. In conclusion, the SCAMP1/miR‐499a‐5p/LMX1A/NLRC5 axis serves as a critical regulator of tumourigenesis and progression of glioma, providing a novel therapeutic strategy for glioma treatment.

## CONFLICT OF INTEREST

The authors declare no conflict of interest.

## AUTHOR CONTRIBUTIONS

YL contributed to the experiment design and implementation, manuscript draft and data analysis. ZZ and YS contributed to the experiment implementation and data analysis. YX conceived and designed the experiments. XR, XL and CY performed the experiments. JZ, SC and ZL analysed the data. ZZ and YS conceived and designed the experiments, performed the experiments and wrote the manuscript. All authors read and approved the final manuscript.

## DATA AVAILABILITY STATEMENT

The data that support the findings of this study are available on request from the corresponding author. The data are not publicly available due to privacy or ethical restrictions.
