# Supplementary figures and images for: Knockdown of LncRNA SCAMP1 suppressed malignant biological behaviours of glioma cells via modulating miR‐499a‐5p/LMX1A/NLRC5 pathway
Source: J Cell Mol Med. 2019 Jun 17;23(8):5048–62. doi: 10.1111/jcmm.14362 (PMC6653555; doi:10.1111/jcmm.14362)

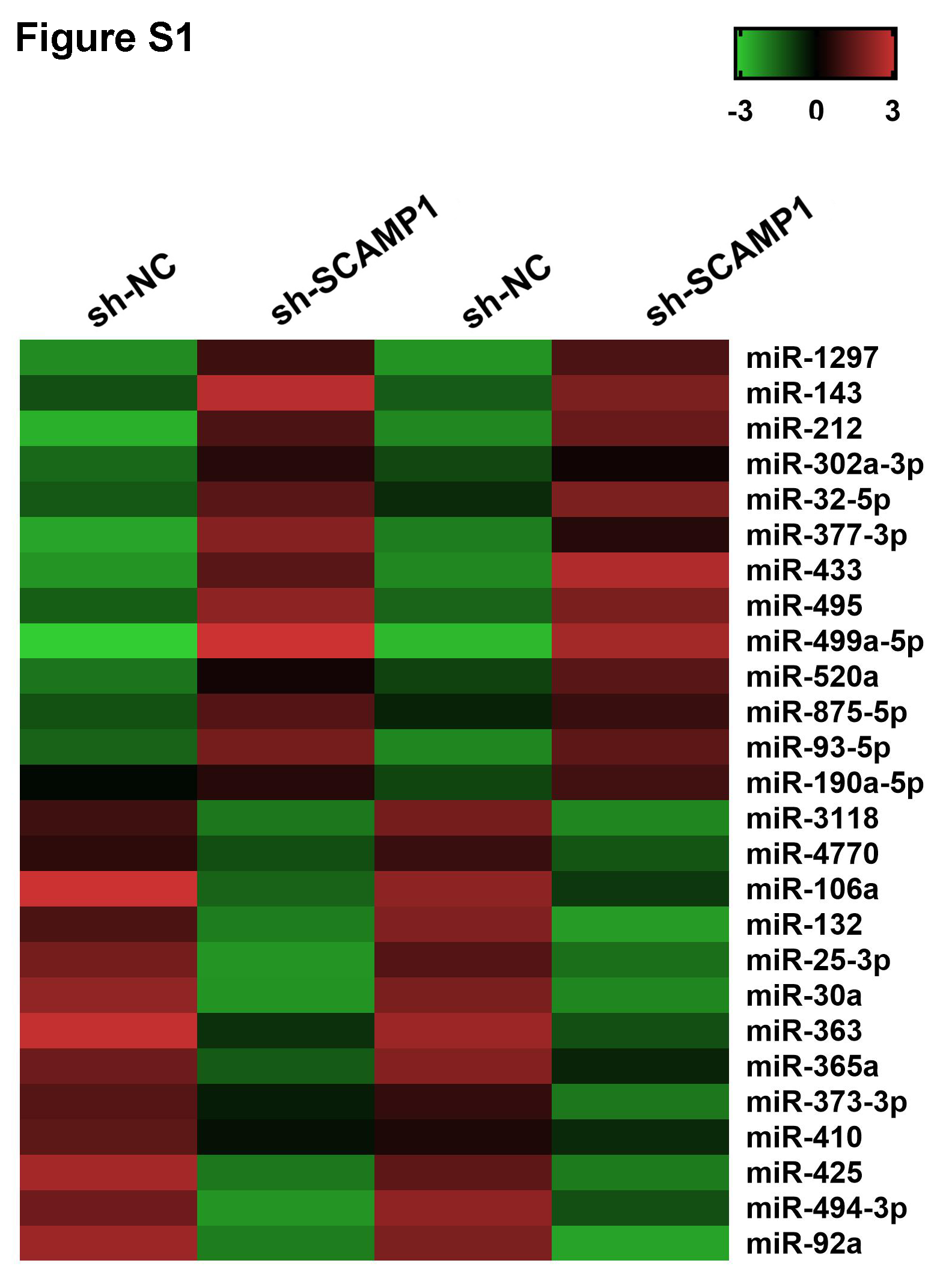

Supplement: Supplementary file 1 [file JCMM-23-5048-s001.tif]

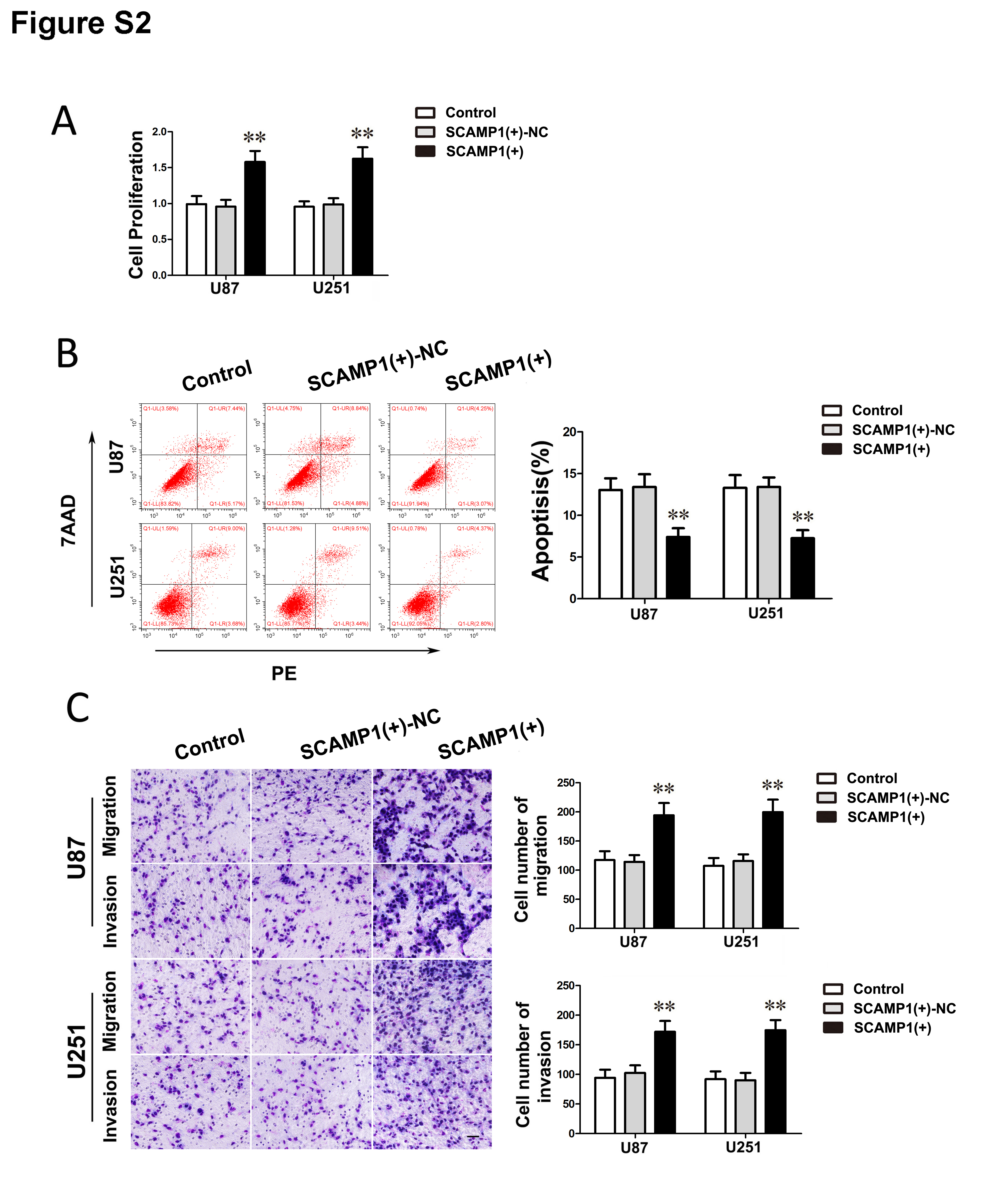

Supplement: Supplementary file 2 [file JCMM-23-5048-s002.tif]

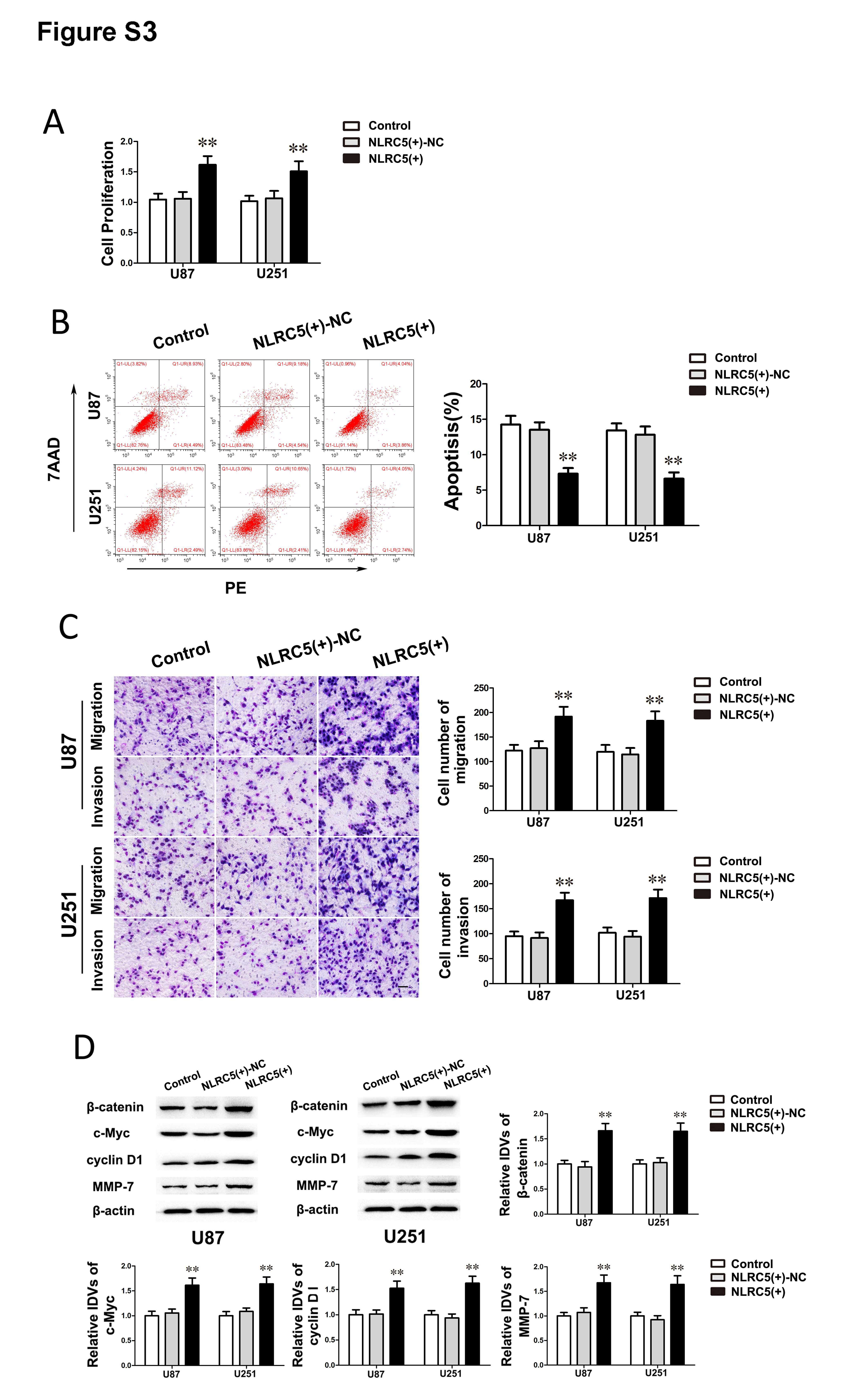

Supplement: Supplementary file 3 [file JCMM-23-5048-s003.tif]
