# Supplementary material for: Knockdown of LncRNA SCAMP1 suppressed malignant biological behaviours of glioma cells via modulating miR‐499a‐5p/LMX1A/NLRC5 pathway
Source: J Cell Mol Med. 2019 Jun 17;23(8):5048–62. doi: 10.1111/jcmm.14362 (PMC6653555; doi:10.1111/jcmm.14362)
Supplement: Supplementary file 4 [file JCMM-23-5048-s004.doc]

| Characteristics | SCAMP1  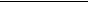 Low High | χ^2^ *P* value |
| --- | --- | --- |
| Age (y) |  | 0.217 0.6410 |
| ≤45 | 4 7 |  |
| ＞45 | 9 11 |  |
| Gender |  | 0.406 0.5241 |
| Male | 5 9 |  |
| Female | 8 9 |  |
| WHO grade  I-II grade | 11 4 | 11.766 0.0006 |
| III-IV grade | 2 14 |  |

**Table S1. Correlation between clinicopathological characteristics and SCAMP1 expression in 31 glioma patients.**

**Table S2. Correlation between clinicopathological characteristics and miR-449a-5p expression in 31 glioma patients.**

| Characteristics | miR-449a-5p  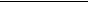Low High | χ^2^ *P* value |
| --- | --- | --- |
| Age (y) |  | 0.940 0.3323 |
| ≤45 | 8 3 |  |
| ＞45 | 11 9 |  |
| Gender |  | 0.185 0.6670 |
| Male | 8 6 |  |
| Female | 11 6 |  |
| WHO grade  I-II grade | 6 10 | 6.348 0.0118 |
| III-IV grade | 13 3 |  |

| Primer or Probe | Gene | Sequence (5'->3') or Assay ID |
| --- | --- | --- |
| Primer | SCAMP1 | F: TGGATGTGAGTGACAGTTC |
|  |  | R: GTGAAAAGTGCTGCTATGA |
|  | LMX1A | F: GAAGGCAAGGACCATAAGCG |
|  | NLRC5 | R: ACCTGGACGACACGGACACT  F: TCTGTTCAGGGTCCAAGGTC  R: TGTGGAGCTGTCTTGTGAGG |
|  | GAPDH | F: AAATCCCATCACCATCTTCCAG |
|  |  | R: TGATGACCCTTTTGGCTCCC |
| Probe | miR-499a-5p | 001352(Applied biosystems) |
|  | U6 | 001973(Applied biosystems) |

**Table S3. Primers and probes used for qRT-PCR.**

**Table S4. Primers used for ChIP assay.**

| Gene | Binding site or Control | Sequence (5'->3') | Product size (bp) |
| --- | --- | --- | --- |
| NLRC5 | PCR1 | F: TTCCTTCTCATCTCCAACCGA | 106 |
|  |  | R: AGTCCGCTATCAGCACATC |  |
|  | PCR2 | F: ACACTTGAACAAATAACCACA | 148 |
|  |  | R: TCACTTGATTTATCTGGACCT |  |
|  | PCR3 | F: GTGGGAATGGATAAGAATGTG | 145 |
|  |  | R: GGATGTCTGGGAGGTGAGT |  |
|  | PCR4 | F: ACATGGAAAGTATTATCATCC | 150 |
|  |  | R: GCAAATAGGAAGGGTAGAGG |  |
