# Supplementary material for: Knockdown of LncRNA SCAMP1 suppressed malignant biological behaviours of glioma cells via modulating miR‐499a‐5p/LMX1A/NLRC5 pathway
Source: J Cell Mol Med. 2019 Jun 17;23(8):5048–62. doi: 10.1111/jcmm.14362 (PMC6653555; doi:10.1111/jcmm.14362)
Supplement: Supplementary file 5 [file JCMM-23-5048-s005.doc]

**Figure S1.** **MiRNAs microarrays data in glioma U87 and U251 cells.** MiRNAs expression profiles were obtained from glioma U87 and U251 cell samples as indicated.

**Figure S2.** **Upregulation of LncRNA SCAMP1 significantly promoted the malignant progression of glioma cells. A.** To evaluate the effects of SCAMP1 overexpression on glioma, cells were divided into three groups: Control, SCAMP1(+)-NC and SCAMP1(+). CCK-8 assay was used to determine the proliferation of U87 and U251 cells treated with SCAMP1 overexpression. **B.** The results of flow cytometry analysis in U87 and U251 cells treated with SCAMP1 overexpression. **C.** Quantification cell number and representative images of migration and invasion in U87 and U251 cells treated with SCAMP1 overexpression were presented. Scale bars represent 40μm. Data is given as mean ± SD (n = 3, each group). ***P*<0.01 vs. SCAMP1(+)-NC group.

**Figure S3.** **Overexpressed NLRC5 enhanced the malignant biological behaviors of glioma cells and upregulated the activity of Wnt/β-catenin signaling pathway. A.** To investigate the effects of NLRC5 overexpression on glioma, cells were divided into three groups: Control, NLRC5(+)-NC and NLRC5(+). CCK-8 assay was used to determine the proliferation of U87 and U251 cells treated with NLRC5 overexpression. **B.** The results of flow cytometry analysis in U87 and U251 cells treated with NLRC5 overexpression. **C.** Quantification cell number and representative images of migration and invasion in U87 and U251 cells treated with NLRC5 overexpression were presented. Scale bars represent 40μm. **D.** Western blot assay showed the expression levels of β-catenin, c-Myc, cyclin D1, MMP-7 in U87 and U251 cells treated with NLRC5 overexpression. Data are presented as the mean ± SD (n = 3, each group). ***P*<0.01 vs. NLRC5(+)-NC group.
